# Reflection and Looking Ahead: A Journey Guided by Purpose

**DOI:** 10.1055/s-0045-1814146

**Published:** 2025-12-31

**Authors:** Krishnamurthy Sridhar

**Affiliations:** 1 Institute of Craniofacial Aesthetic and Plastic Surgery, SIMS Hospital, Chennai, Tamil Nadu, India

*“The greatest surgeries are not performed with the hands alone, but with the heart that beats behind them.”*

## Introduction

Plastic and Reconstructive Surgery has witnessed an extraordinary evolution over the past five decades—a journey I have been privileged to walk. When I began in 1978, we worked with ether anesthesia, tube pedicles, and minimal resources, yet with deep determination and creativity. Cleft lip and palate, hypospadias, gross burn contractures of the neck and hands, industrial injuries, leprosy reconstructions, and posttraumatic deformities formed the backbone of our discipline. Cancrum oris and nares deformities associated with malnourishment have now virtually disappeared, replaced by diabetic foot infections.

At that time, our specialty was widely misunderstood as one focused solely on beautification and perceived to be unnecessary in a country where many lived below the poverty line. We had to firmly establish its true purpose—restoring form, function, and dignity. From those early years to today's era of microvascular surgery, digital design, robotics, artificial intelligence, and three-dimensional (3D) printing, the transformation has been profound. This reflection is not only about technological advancements, but about the philosophy that shaped us: service, compassion, teamwork, and the relentless pursuit of excellence.

We had to educate both the public and the medical community about the importance of early specialist intervention. From introducing the specialty to securing its rightful place in institutions where it was once viewed as nonessential, our collective efforts ultimately demonstrated the value of a trained plastic surgeon capable of intricate, life-changing reconstruction.

## Revolution, Explosion, and Transformation

The early years of my career were defined by surgical creativity rather than technology.

**Fig. 1:**
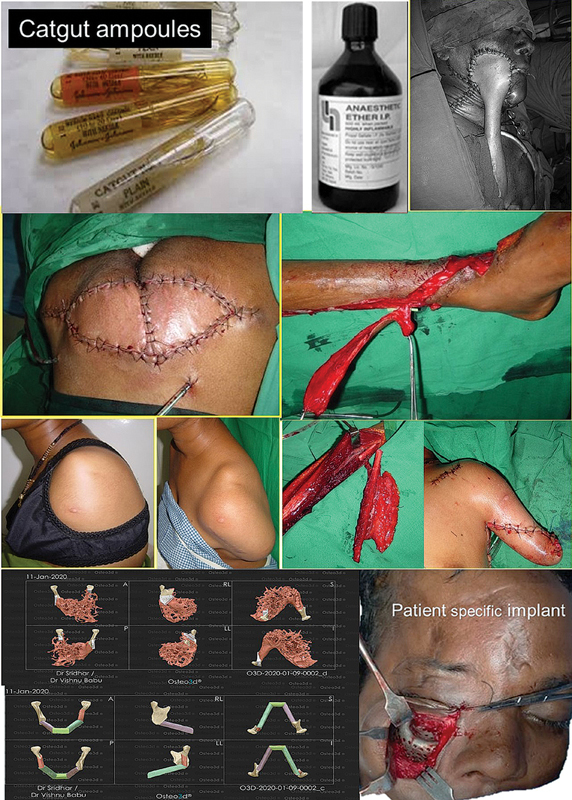
The evolution witnessed through four decades. Top row: cat gut, etherand tube pedicles. Second row: myocutaneous to perforator-based flap. Third row: amelia, tissue expansion, composite flap. Bottom row: computer-assisted planning and patient-specific implant.

Myocutaneous and fasciocutaneous flaps gradually replaced tube pedicles ( Fig. 1 ). The advent of microvascular surgery, however, marked a true revolution. It transformed reconstructive philosophy, enabling precise, reliable free-tissue transfer and shifting practice from regional flaps and staged procedures to single-stage anatomical reconstruction. I was fortunate to be part of the team that performed the first recorded successful reimplantation procedure and to be an early adopter of this new form of reconstruction. The absence of adequate facilities for intracranial craniofacial surgery in the country inspired me to form a collaborative team with neurosurgeons, pioneering craniofacial surgery in southern India.

The decades that followed witnessed an explosion of science and technology—digital planning, 3D printing, advances in imaging, computer assisted navigation biomaterials, instrumentation, and AI-based analysis reshaped surgical possibilities and patient expectations. The operating theater evolved into a convergence of biology, engineering, and computing. Yet beyond revolution and explosion came a deeper transformation—not merely technological, but philosophical.

Health care delivery changed dramatically. Advanced surgical care that was once accessible primarily through government institutions moved into corporate hospitals with state-of-the-art facilities, but at significantly higher costs. Treatments once affordable or free became expensive, pushing patients toward insurance and placing financial strain on families. Subspecialization fragmented the parent discipline, often generating uncertainty and insecurity among colleagues unable to keep pace with rapid change.

## Learning, Adaptation, and Mentorship

In earlier years, access to information was limited. Journals were scarce, books expensive, and the internet nonexistent. Today, knowledge is immediate: surgical videos, lectures, operative demonstrations, and international CME (continuing medical education) programs are available at a touch. Training abroad, once financially and logistically challenging, has now become achievable for many. Yet what we need most today is mentorship—masters who shape not only skills but character, judgment, humility, and discipline. Visiting centers of excellence remains invaluable even now.

## Walking with Progress—One Step at a Time

Much of what I learned in the 1970s is rarely practiced today. If we do not evolve, we lose relevance. Progress demands that we step beyond our comfort zones—gradually extending our boundaries from maxillofacial trauma to orbital and craniofacial reconstruction, orthognathic surgery, custom implants, and endoscopic and robotic procedures. Growth is incremental—never sudden.

## Building and Working with a Team

No individual can excel in every domain of this vast specialty. A strong team with complementary strengths is essential for excellence. Three decades ago, I built a team of four like-minded professionals. Mutual trust, clear communication, defined responsibility, transparent financial structure, and leadership rooted in vision remain the foundations of a successful team.

## Gap Analysis and Finding Purpose

Gap analysis is invaluable within both teams and institutions. As our specialty covers a broad spectrum, opportunities abound—whether in hypospadias care, microvascular oncologic reconstruction, brachial plexus surgery, or maxillofacial services. Identifying a gap reveals possibilities for meaningful contribution. If we lack the skills to address it, we must commit to learning.

## Community Service and Patient-Centric Practice

Ultimately, everything we do must serve society. We are part of the community we treat; our profession finds meaning only when we look beyond ourselves. In 1985, a medical camp organized by the Kanchi Sankaracharya became a turning point in my life. His gentle advice to serve the less privileged inspired me, along with my wife and friends, to establish a medical trust for the slum dwellers of South Chennai. We charged just two rupees for evening consultations, provided free medicines, and ensured complete immunization for children. Over the past forty years, the trust has benefitted more than 800,000 patients.

A chance meeting in 1991 with a classmate involved in the National Leprosy Eradication Programme opened another dimension of service. For nearly fifteen years, I travelled on weekends to a remote village near Erode, performing corrective surgeries for leprosy deformities. This meaningful experience deepened my expertise in tendon transfers and hand dynamics, leading to the development of two new surgical techniques. Compassion, empathy, and patient-centric care must remain the axis around which all innovation revolves. Technology should amplify humanity—not overshadow it.

## Being Part of Administration

Good clinical work cannot thrive within weak organizational systems. When opportunities arise to participate in administration, we must embrace them—because institutional progress depends on informed decision-making. I had the privilege of leading the planning and development of a new hospital, which offered deep insight into hospital operations. The vast nonmedical responsibilities involved in managing health care systems are complex and demand attention to detail. A 2-year course in hospital administration equipped me to later lead not only the hospital but also institutions of medicine, dentistry, and allied health sciences. It enabled a holistic approach to health care and education. Taking responsibilities in our association activities is vital to preserve the integrated parent specialty.

## Be Part of the Evolution, If Not the Axis

Not every new development is superior to what came before, yet progress is refinement—today improves upon yesterday, and tomorrow improves upon today. Tomorrow is going to be technology-driven. We may not do what we do today, we may 3D bioprint tissues and implant them, tissue engineer to replace parts, reconstruct from remote places by telerobotic systems. As long as tissues exist, defects will exist, and as long as defects exist, plastic surgeons will exist, but may perform reconstruction by a tech-driven surgery. We must participate in the evolution of our specialty by evaluating, adopting wisely, and contributing responsibly.

## A Journey Guided by Purpose—A Spiritual Reflection

Looking back, I recognize that my journey has been guided not by personal design but by a higher purpose. Every phase—student, teacher, surgeon, leader, Pro-Vice Chancellor—unfolded through unseen grace. My career has never been only about restoring tissue, but about restoring hope. The true measure of our work lies not in the complexity of operations, but in the light we bring into the lives we touch.

Skill and knowledge are blessings, but compassion is divine. When we serve with sincerity, the universe supports us; when we give without expectation, we become channels of healing. Let us move forward with humility, gratitude, and unwavering resolve—to learn, to innovate, to guide, and to serve.

## Conclusion

As we stand at the crossroads of science, technology, and human need, let us remember that progress begins with belief—belief in ourselves, in our purpose, and in the power of relentless effort. Every great accomplishment in our specialty began as a challenge that someone refused to surrender to. Do not fear change; embrace it. Do not wait for the perfect moment; create it. Step beyond your comfort zone, remain hungry to learn, and be courageous enough to fail—for every setback is a stepping stone to mastery. Build teams, seek mentors, and become one. And let compassion be the compass guiding every decision.

If we touch even one life with sincerity and skill, our journey is already complete. Let us rise each day determined to be better than yesterday—to innovate, to serve, and to leave behind a legacy that makes tomorrow stronger than today.

*“What remains after all achievements fade, is not what we built with skill, but what we touched with love.”*

